# Discrimination of Grape Seeds Using Laser-Induced Breakdown Spectroscopy in Combination with Region Selection and Supervised Classification Methods

**DOI:** 10.3390/foods9020199

**Published:** 2020-02-15

**Authors:** Yong He, Yiying Zhao, Chu Zhang, Yijian Li, Yidan Bao, Fei Liu

**Affiliations:** 1 College of Biosystems Engineering and Food Science, Zhejiang University, 866 Yuhangtang Road, Hangzhou 310058, China; yhe@zju.edu.cn (Y.H.); zhaoyy@zju.edu.cn (Y.Z.); chuzh@zju.edu.cn (C.Z.); yjli@zju.edu.cn (Y.L.); ydbao@zju.edu.cn (Y.B.); 2 Key Laboratory of Spectroscopy Sensing, Ministry of Agriculture and Rural Affairs, Hangzhou 310058, China

**Keywords:** grape seed, laser-induced breakdown spectroscopy, supervised classification, deep learning, region selection

## Abstract

The wine-making industry generates a considerable amount of grape pomace. Grape seeds, as an important part of pomace, are rich in bioactive compounds and can be reutilized to produce useful derivatives. The nutritional properties of grape seeds are largely influenced by the cultivar, which calls for effective identification. In the present work, the spectral profiles of grape seeds belonging to three different cultivars were collected by laser-induced breakdown spectroscopy (LIBS). Three conventional supervised classification methods and a deep learning method, a one-dimensional convolutional neural network (CNN), were applied to establish discriminant models to explore the relationship between spectral responses and cultivar information. Interval partial least squares (*i*PLS) algorithm was successfully used to extract the spectral region (402.74–426.87 nm) relevant for elemental composition in grape seeds. By comparing the discriminant models based on the full spectra and the selected spectral regions, the CNN model based on the full spectra achieved the optimal overall performance, with classification accuracy of 100% and 96.7% for the calibration and prediction sets, respectively. This work demonstrated the reliability of LIBS as a rapid and accurate approach for identifying grape seeds and will assist in the utilization of certain genotypes with desirable nutritional properties essential for production rather than their being discarded as waste.

## 1. Introduction

Grapes (*Vitis vinifera* L.) are one of the largest fruit crops around the world. About 80% of the world’s grapes are used for wine-making [1], which generates grape pomace in an estimated amount of 20%–30% of the initial weight of grapes [2]. Grape seeds account for about 25% of the pomace, but this varies widely among grape cultivars [3]. Due to their high content of bioactive substances, grape seeds have been increasingly used as a natural source for producing food, nutraceutical, cosmetic, and pharmaceutical derivatives [4]. The composition of grape seeds has an impact on their suitability for industrial exploitation, and compositional variation in grape seeds is associated with environmental and viticultural conditions and the cultivar [5,6]. Full utilization of grape seeds of a specific cultivar with desirable properties helps to lower the cost of product making, which indicates the great importance of cultivar discrimination of grape seeds. Discrimination of grape seeds can be implemented via visual inspection by trained personnel, but this approach is rather subjective and laborious. Laser-induced breakdown spectroscopy (LIBS) is a novel laser-based atomic emission spectroscopy that uses laser plasma to achieve multi-element analysis [7]. In a typical LIBS system, a high-energy laser pulse is transmitted to ablate the surface of the sample. Simultaneous vaporization and excitation of the sample result in a higher energy level and form a mixture of atomic and ionic species. As the excited species return to the ground state during de-excitation, the emission lines at characteristic wavelengths are collected by the spectrometer, thus allowing analysis of the elemental composition of the sample [8]. In recent decades, the LIBS technique has become very popular thanks to its rapid analysis, high spatial resolution, and the potential to perform in situ and stand-off analysis [9]. Some attempts have been made to verify the feasibility of the LIBS technique to detect elemental composition and identify specific species of agricultural seeds. Han et al. used LIBS to determine calcium concentrations in different internal tissues of corn seeds, and the detection limit reached 1.05 PPM [10]. Atta et al. identified the emission lines of zinc and iron in wheat by LIBS and performed a qualitative analysis by PCA to identify wheat varieties based on the selected emission lines [11]. Luo et al. used LIBS in the range of 210–480 nm and three pattern recognition algorithms to realize the discrimination of 11 rice species [12]. The average identification rate of the multi-layer perceptron (MLP) neural network was the highest studied, with 100% and 97.9% of the training and test sets, respectively. These researchers explored the feasibility of the LIBS technique for food and agricultural applications. However, to our knowledge, the LIBS technique has not yet been used to trace the cultivars of grape seeds. Grape seeds as sources for producing food, cosmetics, and health-care products are always in powdered form, which is suitable to be pressed into a pellet. Discrimination of grape seeds relies on analysis of the emission lines generated by the interaction of the pellets and the laser pulse.

LIBS spectra are usually complex and noisy signals, and conventional machine learning methods have proven to be effective in dealing with such data [13,14]. On one hand, three traditional supervised classification methods for performing multivariate classification, including support vector machine (SVM), radial basis function neural network (RBFNN), and extreme learning machine (ELM), were used to identify the spectral features of grape seeds in this study. On the other hand, deep learning, which represents a wide class of machine learning methods mostly based on artificial neural networks, has become the most popular topic in the artificial intelligence field. For LIBS data analysis to date, attention has mostly been paid to the implementation of traditional machine learning algorithms [7,11,12], while only a few studies have searched for a way to interpret LIBS data by deep learning approach [15]. To our knowledge, no attempt has been made to evaluate the feasibility of deep learning for discriminating grape seeds. Deep learning and traditional machine learning methods may give different responses to specific applications. This work thus put forward a one-dimensional convolutional neural network (CNN) and compared its predictive capability with the traditional supervised classification methods.

In this work, the LIBS technique was evaluated for the classification of grape seeds of three different cultivars. Specifically, this study was performed to achieve the following objectives: (1) to establish and compare the discriminant models on full spectra by using supervised classification methods including SVM, RBFNN, and ELM; (2) to establish a comparative study of using conventional supervised classification methods and a one-dimensional CNN method; (3) to extract the most relevant spectral region by *i*PLS method; and (4) to build simplified discriminant models for the selected spectral region and compare the performance with the models based on full spectra. The discriminant model exhibiting the highest classification accuracy could be adopted for future uses.

## 2. Materials and Methods

### 2.1. Sample Preparation

Grape seeds including three cultivars (Jufeng, Meirenzhi, and Hongtizi) from a single batch of raw materials were bought from a seed company in Shuyang Pengyuan horticulture farm, Suqian, Jiangsu, China in 2017 for this study due to practical reasons, although multiple batches of raw materials would have provided more information as to the natural variability of the seeds. The three cultivars were recorded as Cultivar I, Cultivar II, and Cultivar III. A total of 590 g seeds of each cultivar was randomly chosen without considering the external features in terms of shape, size, and color. These seeds were cleaned and dried in an oven at 50 °C for 6 h. Every 10 g of seeds constituted a sample and was ground in the grinder (GR150A, Hefei Royalstar Electronic Appliance Co. Ltd., Hefei, Anhui, China) for 60 s. Seed powder was sieved through a 40 mesh sieve. Next, 0.2 g powder of each sample was compressed into a compact pellet by a tablet-press machine (FY-24, SCJS Technology Development Co. Ltd., Tianjin, China) under a pressure of 10 MPa for 60 s. The thickness and the side lengths of the pellet were about 2 mm and 10 mm, respectively. In total, 177 grape seed pellets (59 samples for each cultivar) were obtained and used for LIBS analysis.

### 2.2. LIBS System

**Figure 1:**
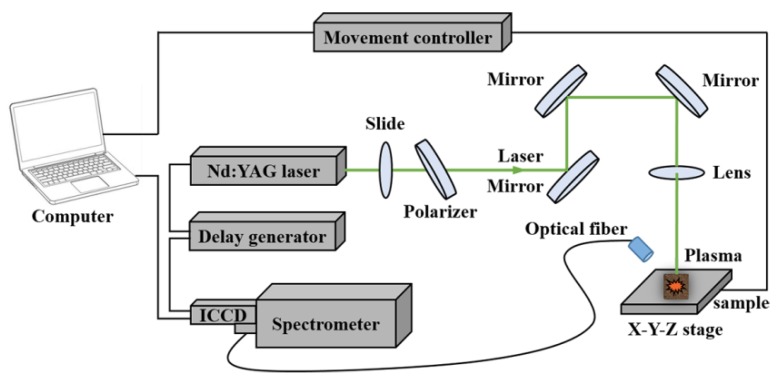
The schematic diagram of the laser-induced breakdown spectroscopy (LIBS) system.

A schematic diagram of the self-built LIBS system used for spectra acquisition is shown in Figure 1. Briefly, the pulse laser was delivered by a Q-switched Nd:YAG nanosecond pulsed laser (Vlite-200, Beamtech Optronics Co. Ltd., Beijing, China) at 532 nm (pulse duration = 8 ns, beam diameter = 7 mm, maximum energy = 200 mJ). The laser energy was adjusted by the combination of a glass slide and a 60° polarizer. The optics (mirrors and lens) were used to guide the beam. A plano-convex lens (*f* = 100 mm) was used to focus the laser beam onto the sample. The emission spectra were then dispersed by the high-resolution Echelle spectrometer (ME5000, Andor Information Technology Ltd., Belfast, UK) and collected by an intensified charge-coupled device (ICCD) camera (DH334, Andor Information Technology Ltd., Belfast, UK) through an optical fiber. The delay time was controlled by a delay generator (DG645, Stanford Research Systems Inc., Sunnyvale, CA, USA). The wavelength range was from 230 nm to 880 nm. Samples were placed on an X-Y-Z movable sample stage that was driven by a movement controller (SC300-1A, Zolix Instruments Co. Ltd., Beijing, China).

To improve the data quality and advance the signal-to-noise (S/N) ratio, some parameters, such as the pulse energy, the delay and integration time, and the gate width, needed to be optimized. In this case, the laser pulse energy was fixed to 60 mJ and the repetition rate was 1 Hz. A 1.5 μs delay time between the laser shot and the radiation collection was set with an integrated time of 10 μs. The gain of the detector was set at 1500. Grape seed pellets were detected directly in the air at atmospheric pressure. In this case, the laser beam was focused 2 mm below the surface of the sample to produce a relatively stable plasma and ablated 4 × 4 array craters. At each position, the spectra with 5 times accumulation were collected and the average spectrum of the 80 spectra was taken as the final spectra for each sample.

### 2.3. Chemometric Method

#### 2.3.1. Principal Component Analysis

Principal component analysis (PCA) is a frequently used multivariate statistical method that works by generating a set of principal components (PCs) that are the linear transformation of the original variables. These new PCs are orthogonal to each other and are sorted according to the explained variance. Generally, the first few PCs explaining most of the total variance are often used for pattern identification [9,16]. PCA is a useful tool to give an easy visualization of the distribution of samples [17,18]. In this work, an overview of the overall data was achieved via PCA by presenting the samples in a newly defined space and grouping them into clusters on the basis of the variance of their corresponding spectra.

#### 2.3.2. Region Selection Method

The original LIBS data contained not only the spectral features relevant for elemental variation in samples, but also the background interference. A reduction in variable space can be carefully conducted by selecting several wavelengths or a range of wavelengths carrying the most useful information for prediction. Interval partial least squares (*i*PLS) is an extension to the traditional PLS, proposed by Nørgaard et al. [19]. In *i*PLS, the whole range of the spectral data is split into several equidistant subintervals within which local PLS models are developed independently [20]. It provides a graphical display of the relevant information of the regression models and permits a comparative analysis among local and global models [21]. The selection criterion of subintervals is mainly based on a validation parameter called root mean squared error of cross-validation (RMSECV). In this study, simple optimization of the best subinterval was carried out in the following steps: (1) divide the whole spectral range into 10 to 30 equidistant subintervals, (2) establish local PLS models on each subinterval with up to 15 latent variables (LVs), and (3) compare the prediction performance of these local PLS models and select the optimal spectral region with the lowest RMSECV among all the subdivisions. Applying *i*PLS not only reduced the computational complexity but also aided in the interpretation of the spectral data.

#### 2.3.3. Classification Methods for Comparison

The selection of chemometric methods depends on the expected application and final objectives. Supervised classification methods are applied to interpret data matrixes composed of objects (input) and classes (target). Such methods aim to build classification models with the capacity to find the relationship between input and target. The three different traditional supervised classification methods used in this work were support vector machine (SVM), radial basis function neural network (RBFNN), and extreme learning machine (ELM). The LIBS spectra were taken as the input (matrix *X*), while the category values of Cultivars I, II, and III were recorded as 1, 2, and 3 as target (vector *Y*), respectively. Two datasets were obtained; the calibration set was used to build the discriminant model, and the prediction set was used to test the model. The test performed on the prediction set reflected the model’s predictive ability on unknown samples. Every third sample was included in the prediction set, starting from Sample 2, while the remaining samples were defined as the calibration set.

SVM is a machine learning algorithm proposed by Vapnik [22], developed based on statistical learning theory. SVM can achieve approximate implementation of structural risk minimization to avoid overfitting [23]. Owing to its surprising classification ability, SVM has been widely used in classification issues [24]. In SVM, the original low dimensional data are first mapped into a higher dimensional space through a nonlinear mapping function. By constructing a hyperplane in the new space, the linear classification of samples can be realized. An RBF kernel was used as the activation function to reduce the computational complexity of the model. The key parameters such as the penalty coefficient (*C*) and kernel function parameter (*γ*) were determined by a grid-search procedure where the range of *C* and *γ* was both set from 2^−8^ to 2^8^.

RBFNN is a simple three-layer feed-forward neural network consisting of an input layer, a hidden layer, and an output layer. In this case, the LIBS spectra were used as the input and were distributed to the hidden layer. RBF was used as the activation function of neurons in the hidden layer. The hidden space was high dimensional, allowing linear separation of samples, and the output layer was a linear combination of the output of neurons in the hidden layer. The spread value was set from 1 to 100 with a step size of 1, and the optimal value was determined according to the highest classification accuracy. RBFNN shows great advantages in fast training and easy initialization [25].

ELM is an efficient learning algorithm for single-hidden layer feed-forward neural networks (SLFNs) proposed by Huang et al. [26], aiming to reach not only the smallest training error but also the smallest norm of weights. This algorithm shows extremely fast learning speed and usually achieves good generalization performance in multi-class classification issues [27]. In this study, the number of the hidden layer neurons was changed from 1 to the size of the calibration set, with the step size defaulted to 1. The optimal number of the hidden layer neurons was determined according to the classification performance in this study.

Deep learning is a very popular method for classification tasks and has been widely used in agricultural engineering. We designed a simple one-dimensional convolutional neural network (CNN) architecture including convolutional layers, max pooling layer, dense layers, SoftMax layers, etc. for 1 pixel spectra inputs. The layers and parameters of the designed CNN are shown in detail in Table 1. The weights of the CNN were initialized using Xavier method. The training was carried out by minimizing the SoftMax cross-entropy loss using the Adam algorithm. In our research, the batch size was set to 20 and the learning rate was set to 0.00001. The CNN model was trained for 1000 epochs with a dropout and weight decay method to avoid overfitting.

### 2.4. Model Evaluation and Software

Classification accuracy was used to evaluate the performance of each supervised classification method, which was defined by calculating the percentage of grape seed samples correctly classified over the total in the calibration and prediction sets. The *i*PLS implementation and SVM, RBFNN, and ELM modeling were conducted using MATLAB R2017b (The Math Works, Natick, MA, USA). CNN was programmed on Python 3 and MXNET framework (Amazon, Seattle, WA, USA). Chi-square test was performed with IBM SPSS statistics V22.0 (IBM Corp., Armonk, NY, USA).

## 3. Results

### 3.1. Preprocessing of Spectra Data

The raw LIBS spectra needed to be preprocessed to minimize the adverse influence of signal variations and random noise caused by environmental conditions, the sample state, and the instrument itself. First, given that noise often existed at the beginning and end of the detection range of the LIBS system, the spectra in the range of 380.01−860.04 nm, including 13,435 wavelengths (variables), were extracted for further analysis. This broad spectral range covered the most spectral characteristics of the samples. Negative spectral intensities generated from noise were transformed to zero by a simple correction. Afterward, wavelet transform (WT) was applied to suppress noise in the raw LIBS spectra while the spectral peaks were kept. The wavelet basis function of Daubechies 6 (db6) with a decomposition level of 3 was used in this case. Minimum and maximum (min-max) normalization was then used to transform the WT-preprocessed data into the range between 0 and 1 by Equation (1):

(1) $$x^{*}=\frac{x-min}{max-min},$$

where x^*^ is the normalized spectra, *x* is the WT-preprocessed spectra, and *min* and *max* are the minimum and maximum values in the WT-preprocessed spectra, respectively.

### 3.2. Principal Component Analysis

**Figure 2:**
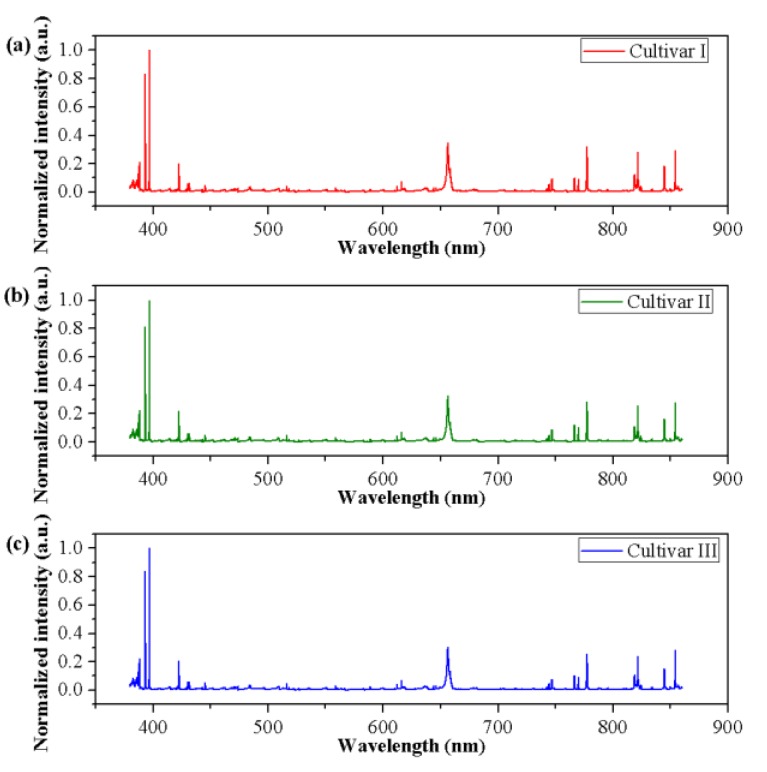
The normalized spectra of grape seeds of (**a**) Cultivar I, (**b**) Cultivar II, and (**c**) Cultivar III. The spectral ranged from 380.01–860.04 nm and the spectral intensities were transformed by min-max normalization.

**Figure 3:**
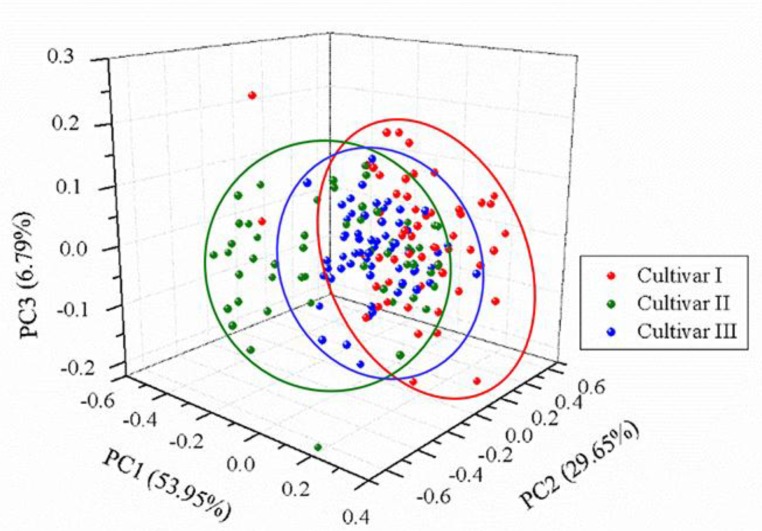
3D score scatter plot of three cultivars based on the first three principal components (PCs). The total variance in the data explained by the first three PCs was 90.39% (53.95%, 29.65%, and 6.79% of PC1, PC2, and PC3, respectively).

The normalized spectra of a randomly selected sample of each cultivar are presented in Figure 2. Visual observation in the normalized spectra showed a high degree of similarity in spectral characteristics among different cultivars. Nevertheless, small differences in peak locations and spectral intensities could be found. To test whether an unsupervised classification was possible, a qualitative analysis method, PCA, was used to explore the differences and patterns among three different cultivars of grape seeds. Results showed that the first three PCs explained 53.95%, 29.65%, and 6.79% of the total variance of the data set, respectively. The variance explained by the first three PCs added up to 90.39%, indicating that most of the spectral information related to samples was involved. Thus, the new coordinate space was defined by the scores of the first three PCs. The 3D score scatter plot (*X*-axis: PC1, *Y*-axis: PC2, and *Z*-axis: PC3) is presented in Figure 3 and each cultivar is shown with a different color for better visualization. Samples of each cultivar grouped but overlapping among different cultivars could be observed, and several samples were away from the cluster center. Overall, PCA could provide an overview of sample distribution but could not provide sufficiently clear discrimination. Therefore, other multivariate methods should be considered.

### 3.3. Discriminant Models on the Full Spectra

Traditional machine learning methods including SVM, RBFNN, and ELM, and a one-dimensional CNN architecture were used to establish calibration models on the normalized spectra. The discriminant results are shown in Table 2 and chi-square test results for the prediction sets are shown in Table 3. Discriminant models based on SVM, RBFNN, and ELM methods showed satisfactory results, with calibration accuracies over 96.6% and prediction accuracies over 86.7%. In terms of different cultivars, the classification performance of different discriminant models varied, within which the grape seeds of Cultivar III were generally identified with the highest classification accuracy. This indicated that grape seeds from different origins could influence the discrimination results. The classification performance of the RBFNN model outperformed the other two models based on traditional supervised classification methods, but there was no significant difference between them. Among all the discriminant models, the CNN model achieved the best performance, with classification accuracy for the calibration and prediction sets of 100% and 96.7%, respectively. Additionally, the CNN model was significantly better than the SVM model (*p*-value < 0.05). This indicated the superiority of applying CNN in grape seed discrimination in the original full-spectra form because of its ability to automatically learn deep spectral feature.

### 3.4. Spectral Region Selection by Interval Partial Least Squares

**Figure 4:**
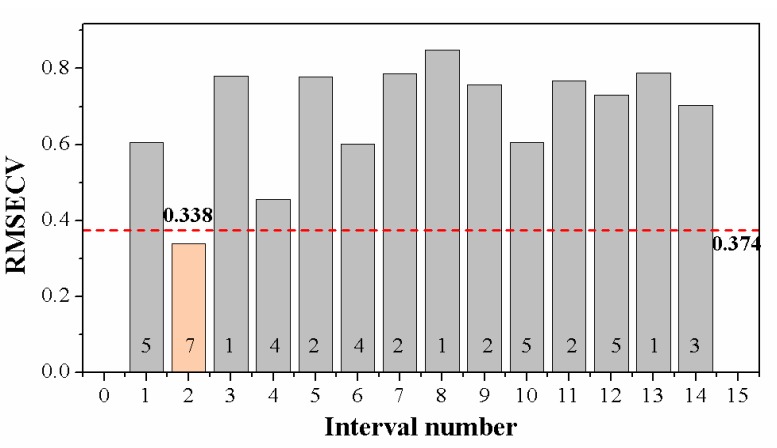
Performance of local partial least squares (PLS) models with 14 equidistant subintervals in 402.74–426.87 nm. The numbers above the *X*-axis are the optimal latent variables (LVs) in corresponding local PLS models. The lowest root mean squared error of cross-validation (RMSECV) of the second local PLS model was 0.338 (LVs = 7). The red dash-dot line represents the RMSECV of the global model (LVs = 11).

The full spectra contained 13,435 variables, resulting in high modeling complexity and computational cost. Some spectral features related to elemental composition in samples, while some were background or instrumental information that was unfavorable for identifying grape seeds. Therefore, appropriate methods were used to select a relevant spectral region and remove the useless background information. A region selection algorithm, *i*PLS, was used in this study. Local PLS models were developed independently for each subinterval, and the performance evaluated by RMSECV is shown in Figure 4. When the whole spectral range was split into 14 subintervals, the local PLS model developed on the second subinterval with seven LVs yielded the best results. Its value of RMSECV was the lowest, even lower than the global PLS model, indicating that applying *i*PLS not only reduced the dimension of the input matrix but also helped to promote the robustness of the discriminant model. The subinterval chosen by *i*PLS covered the spectral range from 402.74 nm to 426.87 nm. The number of wavelengths (variables) in this local PLS model was reduced to 960, which accounted for only 7.1% of the original variables.

**Figure 5:**
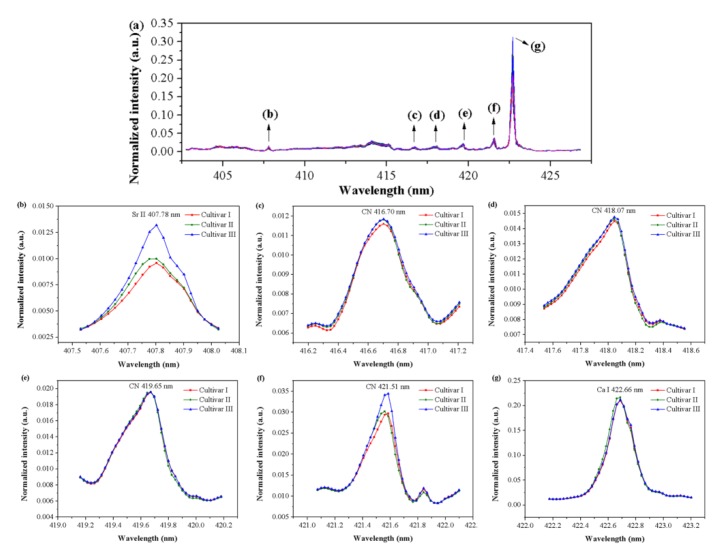
(**a**) The spectral region (402.74–426.87 nm) selected by interval partial least squares (*i*PLS) and the average spectra near emission lines at (**b**) 407.78 nm, (**c**) 416.70 nm, (**d**) 418.07 nm, (**e**) 419.65 nm, (**f**) 421.51 nm, and (**g**) 422.66 nm. The spectra near the emission lines represent the average spectra of each cultivar in the calibration set.

The spectra selected by *i*PLS for three cultivars are shown in Figure 5a. According to the Atomic Spectra Database (ASD) drafted by National Institute of Standards and Technology (NIST) [28] and previous studies, the main spectral peaks observed within 402.74–426.87 nm were assigned to specific emission lines of some elements, and a comparison of the average spectra near these emission lines is presented in Figure 5b–g. The spectral peak at 407.78 nm was related to the ionic emission line of Sr II [28]. A series of spectral peaks observed at 416.70, 418.07, 419.65, and 421.51 nm corresponded to the molecule bands of CN [29], which was a reflection of the composition of organic matters in grape seed samples. The spectral peak at 422.66 nm was related to the atomic emission line of Ca I [28].

### 3.5. Discriminant Models on the Selected Spectral Region

By applying the *i*PLS method to select the most important spectral region, the number of input variables was reduced to 960, which helped to build more stable and simpler models. SVM, RBFNN, ELM, and CNN methods were used to establish discriminant models in this spectral range and the results are summarized in Table 4. Chi-square test results for the prediction sets are shown in Table 5. The classification performance for each cultivar depended on the discriminant methods used. The three traditional discriminant models were all based on nonlinear classification, yielding in all cases calibration accuracies higher than 96% and prediction accuracies over 78%. RBFNN was the most sensitive to wavelength reduction with the largest drop in classification accuracy for the prediction set, and its classification performance was significantly worse than the other models (all *p*-value < 0.05). When compared with the full-spectra-based model, the classification accuracies of SVM and ELM models based on the selected spectral region were slightly higher than the corresponding full-spectra prediction models. The SVM model obtained the best results among all the discriminant models based on the preferred spectral region, although there was no significant difference between the SVM and ELM models or the SVM and CNN models. In general, running the simplified SVM model with a narrow wavelength region could remarkably reduce the dimension of input variables, thereby decreasing the computational complexity to a large extent. On the other hand, however, a slight decrease in the prediction accuracy was observed in the CNN model developed on the selected spectral region when compared to the original model. Owing to the loss of some useful spectral information, the comparatively worse performance was reasonable. It should be noted that the CNN model based on the full spectra exhibited better predictive ability than all the discriminant models developed using the conventional supervised classification methods based either on full spectral data or the selected spectral region, except for the simplified SVM model which achieved consistent performance. This phenomenon proved the excellent ability of CNN to extract discriminative features using the original spectral information, while the traditional supervised classification methods such as SVM and ELM relied more on the artificial extraction of important spectral features to avoid distractions from the useless background or instrumental interference.

## 4. Discussion

Some researchers have explored the feasibility of LIBS for seed identification and composition prediction [30,31]. These works have made an emphasis on performing qualitative or quantitative analysis based on selecting an optimum combination of characteristics emission lines by using single variable selection algorithms. However, LIBS spectra always contain tens of thousands of informative variables, which greatly increase the computational complexity of the traditional single variable selection process [32]. Instead, we investigated the effectiveness of using a region selection method of *i*PLS to extract the important spectral range. Compared with single variable selection algorithms, the conduction of region selection showed superiority in eliminating the influence caused by the fluctuation of characteristic emission lines of target elements during data acquisition. After implementation of the *i*PLS algorithm, the huge number of variables was reduced into a continuous spectral region and the intensities of main spectral peaks corresponding to molecule bands of CN varied among grape seeds of different cultivars, which was consistent with a previous study [31]. The intensities of CN bands could reflect the variance in organic matters such as protein, sugar, and fat among different cultivars, which might be in response to the differences in physiological metabolism and could be studied in future work.

In addition to the utility of classical supervised classification methods combined with manual characteristic spectral region selection in recognizing sample features, we explored the feasibility of applying deep learning in differentiating grape seeds and compared its effectiveness with a later prediction based on manually selected spectral region. We chose a one-dimensional CNN which could automatically learn to extract features through multiple convolutional layers as the classification architecture. In some previous studies in spectroscopic tasks, deep learning methods routinely outperformed other traditional machine learning methods [33,34]. In terms of deep learning in LIBS applications, Zhao et al. attempted to identify different concentrations of lead in soil where tobacco was grown to absorb the contamination [15]. The deep belief network (DBN) classifier performed better than the SVM and partial least squares-discriminant analysis (PLS-DA) models for samples contaminated for two and four weeks. Similarly, in our study, the CNN model based on the full spectra achieved the best performance among all the discriminant models, yielding a classification accuracy of 100% and 96.7% for the calibration and prediction sets, respectively. The simplified SVM model based on the optimal spectral region also achieved consistent performance, which would help to provide accurate identification of grape seeds in short period time. However, the detection system needed to be further updated when new samples showed up. In this regard, the CNN model with superior self-study ability in analyzing deep spectral features from the original data would be more suitable for developing a real-time monitoring system to meet the demand for automatic detection in the modern industry. More seed samples from more origins could be collected in the future to build a more robust model and to improve the classification accuracy. This work also indicated that the high-dimensional LIBS data might be a great resource to train CNN and to fully exploit its advantages.

## 5. Conclusions

In this study, we studied whether the LIBS technique combined with chemometric methods including region selection and supervised classification methods could be used as a novel way to identify grape seeds. The comparison among the discriminant models based on the full spectra and the spectral region selected by *i*PLS indicated that appropriate chemometric methods should be considered for the specific problem to be solved. Additionally, by applying region selection methods, spectral characteristics of samples could be extracted to reduce the computational time while promoting the model’s identification ability. The CNN model which automatically learnt deep features from the full spectra also obtained satisfactory performance. Combining the advantages of rapid data acquisition of LIBS and the self-study capability of CNN, an on-line detection system for grape seeds as well as other agricultural seeds can be developed. This research provides fundamental guidance for identifying the specific cultivars of grape seeds for future use in producing food, cosmetics, and healthcare products rather than discarding them as waste. Without complex sample treatment or consumption of chemical reagents, the LIBS technique allows rapid spectra acquisition with valuable elemental information, and deep learning achieved better interpretation of the spectral data. The methodology shows great potential for the rapid discrimination of grape seeds.

## Figures and Tables

**Table 1:** Layers and parameters of the designed convolutional neural network (CNN) architecture.

Layers	Parameters	Activation	Additional Processing
Convolution-1D (1)	Kernel number = 32, Kernel size = 3, Strides = 1	ReLU	Batch normalization
Max pooling	Size = 2, Strides = 2	-	-
Convolution-1D (2)	Kernel number = 16, Kernel size = 3, Strides = 1	-	Batch normalization
Dense (1)	Neurons = 512	ReLU	Batch normalization, Dropout (0.5)
Dense (2)	Neurons = 32	ReLU	Batch normalization, Dropout (0.2)
Dense (3)	Neurons = 3	ReLU	-
SoftMax	-	-	-

**Table 2:** The discriminant results of support vector machine (SVM), radial basis function neural network (RBFNN), extreme learning machine (ELM), and convolutional neural network (CNN) models on the full spectra.

Models	Parameter ^1^	Calibration Set					Prediction Set			
		-	1	2	3	Accuracy	1	2	3	Accuracy
**SVM**	(9.1896, 9.1896)	1	39	0	0	100%	15	4	1	75.0%
		2	0	39	0	100%	2	18	0	90.0%
		3	0	0	39	100%	0	1	19	95.0%
		Total	-	-	-	100%	-	-	-	86.7%
**RBFNN**	2	1	39	0	0	100%	20	0	0	100%
		2	0	39	0	100%	3	17	0	85.0%
		3	0	0	39	100%	0	0	20	100%
		Total	-	-	-	100%	-	-	-	95.0%
**ELM**	48	1	39	0	0	100%	17	2	1	85.0%
		2	2	35	2	89.7%	2	18	0	90.0%
		3	0	0	39	100%	1	1	18	90.0%
		Total	-	-	-	96.6%	-	-	-	88.3%
**CNN**	Seen in Table 1	1	39	0	0	100%	20	0	0	100%
		2	0	39	0	100%	2	18	0	90.0%
		3	0	0	39	100%	0	0	20	100%
		Total	-	-	-	100%	-	-	-	96.7%

^1^ The parameters of the SVM model were the penalty coefficient (*C*) and kernel function parameter (*γ*); the parameter of the RBFNN model is the spread value; the parameter of the ELM model was the number of nodes in the hidden layer; the parameters of CNN can be found in Table 1.

**Table 3:** Chi-square test for the prediction results of support vector machine (SVM), radial basis function neural network (RBFNN), extreme learning machine (ELM), and convolutional neural network (CNN) models on the full spectra.

-	*p*-Value ^1^
SVM vs. RBFNN	0.114
SVM vs. ELM	0.783
SVM vs. CNN	0.048
RBFNN vs. ELM	0.186
RBFNN vs. CNN	1.000
ELM vs. CNN	0.163

^1^ A *p*-value less than 0.05 was considered statistically significant in this study.

**Table 4:** The discriminant results of support vector machine (SVM), radial basis function neural network (RBFNN), extreme learning machine (ELM), and convolutional neural network (CNN) models on the selected spectral region.

Models	Parameter ^1^	Calibration Set					Prediction Set			
		-	1	2	3	Accuracy	1	2	3	Accuracy
SVM	(147.0334, 27.8576)	1	39	0	0	100%	18	2	0	90.0%
		2	0	39	0	100%	0	20	0	100%
		3	0	0	39	100%	0	0	20	100%
		Total	-	-	-	100%	-	-	-	96.7%
RBFNN	3	1	39	0	0	100%	14	6	0	70.0%
		2	0	39	0	100%	4	16	0	80.0%
		3	0	0	39	100%	0	3	17	85.0%
		Total	-	-	-	100%	-	-	-	78.3%
ELM	48	1	39	0	0	100%	20	0	0	100%
		2	2	35	2	89.7%	1	17	2	85.0%
		3	0	0	39	100%	0	1	19	95.0%
		Total	-	-	-	96.6%	-	-	-	93.3%
CNN	Seen in Table 1	1	39	0	0	100%	18	2	0	90.0%
		2	0	39	0	100%	1	19	0	95.0%
		3	0	0	39	100%	0	0	20	100.0%
		Total	-	-	-	100%	-	-	-	95.0%

^1^ The parameters of the SVM model were the penalty coefficient (*C*) and kernel function parameter (*γ*); the parameter of the RBFNN model was the spread value; the parameter of the ELM model was the number of nodes in the hidden layer; the parameters of CNN can be found in Table 1.

**Table 5:** Chi-square test for the prediction results of support vector machine (SVM), radial basis function neural network (RBFNN), extreme learning machine (ELM), and convolutional neural network (CNN) models on the full spectra.

-	*p*-Value ^1^
SVM vs. RBFNN	0.002
SVM vs. ELM	0.679
SVM vs. CNN	1.000
RBFNN vs. ELM	0.018
RBFNN vs. CNN	0.007
ELM vs. CNN	1.000

^1^ A *p*-value less than 0.05 was considered statistically significant in this study.
